# Effect of Sichuan Pepper (*Zanthoxylum* genus) Addition on Flavor Profile in Fermented Ciba Chili (*Capsicum* genus) Using GC-IMS Combined with E-Nose and E-Tongue

**DOI:** 10.3390/molecules28155884

**Published:** 2023-08-04

**Authors:** Baozhu Wu, Chenglin Zhu, Jing Deng, Ping Dong, Yiling Xiong, Huachang Wu

**Affiliations:** 1 College of Food and Biological Engineering, Chengdu University, Chengdu 610106, China; wubaozhu@stu.cdu.edu.cn (B.W.); dj3930590@sina.com (J.D.); dpmumu@sina.com (P.D.); xiong98920@sina.cn (Y.X.); 2 Cuisine Science Key Laboratory of Sichuan Province, Sichuan Tourism University, Chengdu 610100, China; 3 College of Food Science and Technology, Southwest Minzu University, Chengdu 610041, China; chenglin.zhu@swun.edu.cn

**Keywords:** Ciba chili, Sichuan pepper, E-nose, E-tongue, GC-IMS

## Abstract

This study examined the flavor profiles of fermented Ciba chili, comparing samples with Sichuan pepper (HJ) to those without Sichuan pepper (CK), using three analytical techniques: E-tongue, E-nose, and gas chromatography–ion mobility spectrometry (GC-IMS). The results obtained from the E-tongue and E-nose exhibited a clear difference in taste and flavor between CK and HJ. In detail, CK mainly exhibited a sour flavor profile, whereas HJ displayed an intricate and rich flavor. The HS-GC-IMS results identified a total of 60 compounds in the samples, with terpenes, alcohols, and esters being the primary volatile flavor compounds. Additionally, *Zanthoxylum* was found to significantly enhance the concentration of these compounds in fermented Ciba chili. Through robust principal component analysis (rPCA), 17 distinct flavor compounds were selected. Correlation analysis revealed that most terpenes exhibited positive correlations with LY2/LG, LY2/gCT1, LY2/Gct, LY2/G, LY2/Gh, and terpenes were found in higher concentrations in HJ. This study contributes a theoretical basis and provides data support for optimizing the fermentation process and elucidating the underlying mechanism of characteristic aroma formation in Ciba chili after fermentation.

## 1. Introduction

Pepper, belonging to the *Capsicum* genus, is a widely consumed vegetable, condiment, and spice with a long history of usage spanning centuries across various regions of the world [1]. In 2021, the worldwide production of fresh chilies and peppers amounted to 71.31 million tons, wherein China emerged as the second-largest producer, contributing around 10.17% of the total output [2]. Southwest China serves as the main region for chili consumption, where a substantial quantity of chili is consumed annually. A notable portion of the chilies is processed into various chili products, including Paojiao [3], Zha-chili [4], and Ciba chili. Ciba chili is a distinct type of chili product originating from Guizhou, Yunnan, and Sichuan provinces. It is prepared by cleaning, chopping, and blending dried chilies with various ingredients. However, Ciba chili lacks aroma and is characterized by a less pronounced spiciness due to the inherent properties of the pepper itself. To enhance its flavor and reduce pungency, fermentation has been employed as an additional step in the preparation process. Furthermore, fermentation serves as a simple yet effective method for extending the shelf-life of perishable raw materials [5]. In the Sichuan province of China, chilies (*Capsicum* genus) are commonly combined with Sichuan pepper (*Zanthoxylum* genus) to create the distinctive mala flavor, which translates to “numbing and spicy” [6]. Sichuan pepper, also known as “Huajiao”, is a quintessential element of Sichuan cuisine and is used in dishes such as spicy rabbit head and hot pot. Therefore, when making Ciba chili, it is customary to add a specific proportion of Sichuan pepper to enhance its flavor characteristics. Extensive research has been conducted on the flavor compounds of Sichuan pepper, with a focus on unsaturated alkyl amides known as sanshools. Additionally, volatile oils such as linalyl acetate, linalool, and limonene are present [7]. However, the influence of Sichuan pepper on the flavor of the processed Ciba chili product remains to be studied.

Flavor plays a pivotal role in determining the quality of food, encompassing both aroma and taste [8]. E-sense technology, comprising electronic nose (E-nose) and electronic tongue (E-tongue) sensors, provides a digital means to simulate human olfaction and taste, enabling rapid and accurate evaluation of food flavors [9]. Gas chromatography–ion mobility spectrometry (GC-IMS), on the other hand, is a powerful technique for the separation and sensitive detection of volatile organic compounds. It offers advantages such as swift response, high sensitivity, ease of operation, and cost-effectiveness [10]. These three technologies are commonly combined and extensively applied in the food industry, ranging from detecting food adulteration to optimizing processing techniques and selecting suitable storage conditions [11,12,13,14]. Existing research on fermented pepper products primarily focuses on flavor analysis, microbial diversity analysis, and the relationship between key flavors and microorganisms. For instance, Xu et al., identified *Rosenbergiella* and *Staphylococcus* as the dominant bacterial genera, with *Hyphopichia* and *Kodamaea* being the most abundant fungi in fermented chili pepper [15]. Zhang et al., found that the influence of brine on organic acids, sugars, and aroma was more significant than that of containers, while the production of free amino acids was more influenced by containers rather than brines [16]. Xu et al., discovered a strong correlation between *Aspergillus*, *Bacillus*, *Brachybacterium*, *Microbacterium*, *Staphylococcus*, and the formation of flavor in red fermented chili [17]. Chen et al., observed a significant negative correlation between the presence of Lactobacillus and salinity, while salinity exhibited a positive correlation with species richness and evenness in fermented chili [18].

In this study, two batches of fermented Ciba chili, one with Sichuan pepper and one without, were prepared using natural fermentation for a duration of 35 days. The objective of this research was threefold. Firstly, the intensity of each flavor quality was assessed by integrating data from the E-tongue and E-nose systems. Secondly, a qualitative and quantitative analysis of flavor compounds was conducted using GC-IMS. Thirdly, a correlation analysis was performed to investigate the relationship between the flavor profile and the significant sensors. The primary goal of this study was to gain new insights into the variation in aroma profiles of Ciba chili and elucidate the underlying mechanisms contributing to the formation of its characteristic aroma after fermentation.

## 2. Results

### 2.1. E-Tongue Analysis

**Figure 1:**
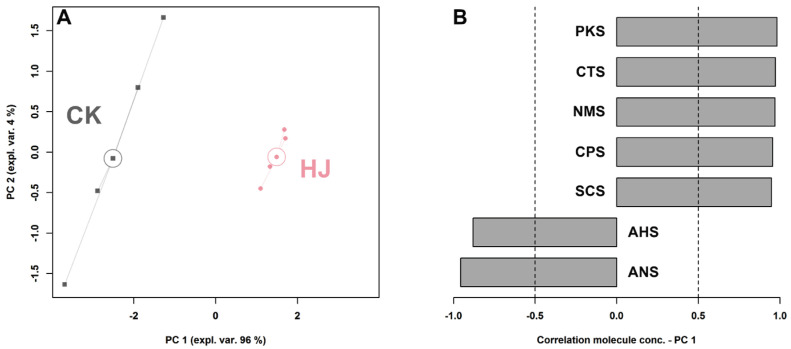
An rPCA model was constructed using the E-tongue response data, and the results are presented in the score plot (**A**). Additionally, a Pearson correlation plot of the loading was generated (**B**).

To discern the overall taste disparity between CK and HJ, an electronic tongue was employed. Six of the sensors gave a significantly different response when analyzing the two groups (*p* < 0.05). In order to capture the overall trend of these sensors, their response values were utilized to construct a robust principal component analysis (rPCA) model. As illustrated in Figure 1A, PC1 accounted for 96% of the variance and effectively captured the overall information of the samples, indicating a successful differentiation between CK and HJ based on PC1. When comparing the two groups, significant differences (*p* < 0.05) were observed in the responses of all seven sensors. Specifically, HJ exhibited higher response values in PKS, CTS, NMS, CPS, and SCS sensors, while displaying lower response values in AHS and ANS sensors (Figure 1B). This suggests that the taste profile of CK is characterized by a sour flavor, whereas the addition of Sichuan pepper to fermented Ciba chili enhances its savory and umami characteristics.

### 2.2. E-Nose Analysis

**Figure 2:**
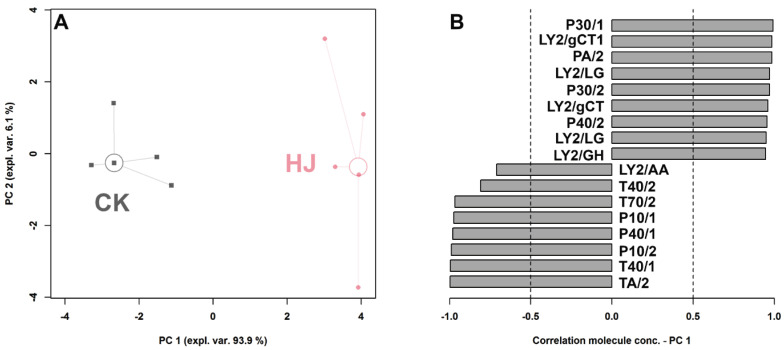
An rPCA model was constructed using the E-nose response data, and the results are presented in the score plot (**A**). Additionally, a Pearson correlation plot of the loading was generated (**B**).

The E-nose is an intelligent sensory technology capable of discriminating between samples and providing a comprehensive flavor profile. As shown in Figure 2A,B, PC1 accounts for 93.9% of the total variance and clearly distinguishes between HJ and CK, indicating significant differences in their flavor profiles. With the exception of T30/1, all other sensors exhibit significant differences (*p* < 0.05). In CK, the sensors LY2/AA, T40/2, T70/2, P10/1, P40/1, P10/2, T40/1, and TA/2 demonstrate significantly higher response values, whereas, in HJ, the response values of P30/1, LY2/gCT1, PA/2, LY2/LG, P30/2, LY2/gCT, P40/2, LY2/LG, and LY2/GH are higher. Based on the sensor performance of the electronic nose, it is evident that CK exhibits a mild aroma profile, while HJ yields a more intense and complex aroma.

### 2.3. GC-IMS Analysis

**Figure 3:**
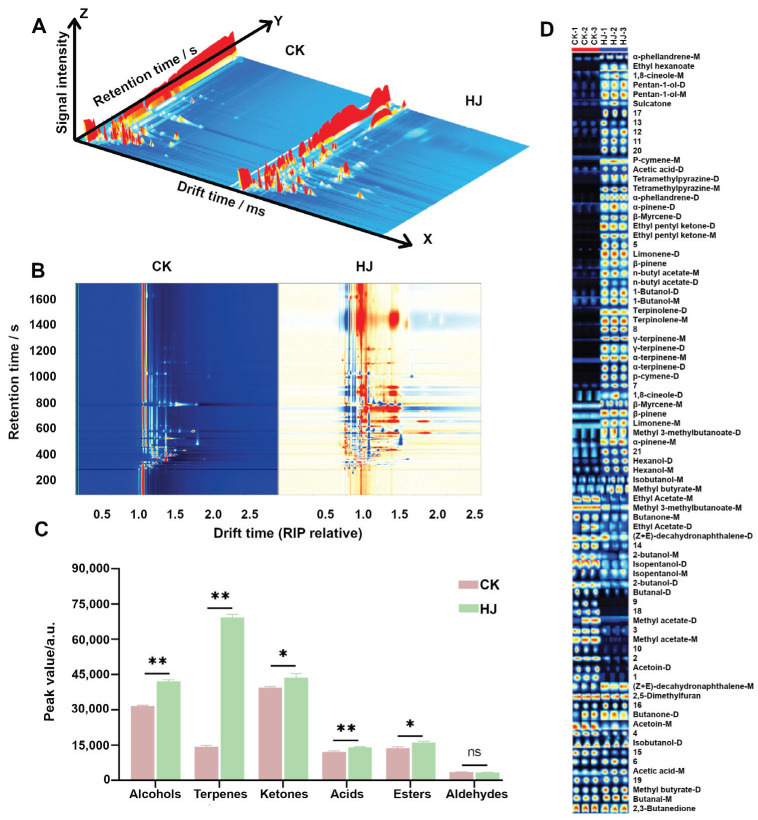
GC-IMS observations were conducted on fermented CK and HJ samples. (**A**) Three-dimensional representation of the observations is shown. (**B**) A bird’s eye view representation is provided, where the spectra from CK are used as a reference, and the spectra from HJ are depicted as differences compared to CK samples. (**C**) The peak volume of volatile flavor substances in CK and HJ is presented. (**D**) The volatile fingerprint of CK and HJ is displayed. ** indicates extremely significant difference (*p* < 0.01), * indicates significant difference (*p* < 0.05), ns indicates no signifcant difference (*p* > 0.05).

GC-IMS is an innovative technology known for its rapid detection and simple pretreatment, offering a specific flavor profile that complements the capabilities of the electronic nose. In the 3D representation shown in Figure 3A, clear visual distinctions can be observed between samples from CK and HJ across a wide range of the GC-IMS spectrum. To facilitate the comparison of volatile flavor substances between CK and HJ, we utilized the topographical plot of CK as a reference and subtracted the topographical plots of HJ from it. In this representation, the deduction of white color indicates an equivalent concentration of volatile flavor substances in both CK and HJ. Conversely, the presence of a red dot signifies a higher concentration of a particular substance compared to the reference, while the blue color denotes a lower concentration. As depicted in Figure 3B, a greater number of red dots can be observed, indicating discernible differences in volatile flavor substances between CK and HJ. Furthermore, it was found that the total content of volatile flavor substances was higher in HJ than in CK.

A total of 81 peaks were detected in CK and HJ, and 60 substances (including monomers and dimers) were identified. These substances comprised 14 alcohols, 2 aldehydes, 2 acids, 11 esters, 16 terpenes, and 9 ketones. The relevant information about each of them is provided in Table 1.The qualitative analysis of volatile flavor compounds in CK and HJ is presented in Figure 3C. There was no statistically significant difference in aldehyde content between CK and HJ (*p* > 0.05). However, alcohols, acids, esters, terpenes, and ketones in HJ were found to be significantly higher compared to CK (*p* < 0.05). Acids exhibited the highest content of volatile flavor substances in both CK and HJ, followed by terpenes and ketones. The samples contained a single organic acid, namely acetic acid. Natural terpenes and their derivatives possess diverse aroma characteristics, making them significant contributors to food flavor. In the samples, a total of eight terpenes were identified, namely α-phellandrene, α-pinene, α-terpinene, β-pinene, γ-terpinene, limonene, β-myrcene, and terpinolene. The concentration of these compounds was higher in HJ than in CK, suggesting that the addition of *Zanthoxylum* promotes the formation of terpenes during Ciba chili fermentation. Nine ketones were detected in the samples, among which sulcatone, ethyl pentyl ketone, and acetoin exhibited significant differences between CK and HJ. The content of these ketones was significantly higher in HJ than in CK (*p* < 0.05). Aldehydes, known for their lower threshold, play a significant role in the flavor profile of Ciba chili during fermentation. Only one aldehyde, butanal, was detected in the samples, with a significantly higher content in CK compared to HJ (*p* < 0.05). Seven alcohols were detected in the samples, with 2-butanol, isobutanol, and isopentanol exhibiting higher levels in CK, while 1,8-cineole, hexanol, pentan-1-ol, and 1-butanol exhibited higher levels in HJ. Additionally, six esters were detected in the samples, among which ethyl acetate, methyl acetate, n-butyl acetate, ethyl hexanoate, and methyl 3-methylbutanoate exhibited significant differences between CK and HJ. Specifically, ethyl acetate and methyl acetate had higher content in CK, while n-butyl acetate, ethyl hexanoate, and methyl 3-methylbutanoate had higher content in HJ. To further differentiate the volatile flavor substances between CK and HJ, all detected compounds in the GC-IMS spectra were selected to generate fingerprints using the Gallery Plot plug-in, as illustrated in Figure 3D. Each column in the gallery plot represents the complete signal peak of a sample, while each row displays the signal intensity of identical compounds found in different samples. The changes in volatile compounds can be clearly seen through the fingerprint, among which butanal-D, methyl acetate-D/M, and acetoin-D are the characteristic flavor substances in CK, while the characteristic flavor substances in HJ are mainly terpenes, such as α-phellandrene-M/D, ethyl hexanoate, 1,8-cineole-M, pentan-1-ol-D/M, sulcatone, p-cymene-M, and acetic acid-D, tetramethylpyrazine-D/M, α-pinene-D, bete-myrcene-D, ethyl pentyl ketone-D/M, limonene-D, β-pinene, n-butyl acetate-M/D, 1-butanol-D/M, terpinolene-D/M, γ-terpinene-M/D, α-terpine-M/D, and p-cymene-D. The addition of prickly ash will also make some substances change. For example, ethyl acetate-M/D, methyl 3-methylbutanoate-M, butanone-M, (Z + E)-decahydronaphthalene-D, 2-butanol-M/D, isopentanol-M/D, 2-butanol-D will decrease with the addition of *zanthoxylum*, while 1,8-cineole-D, bete-myrcene-M, β-pinene, limonene-M, methyl 3-methylbutanoate-D, α-pinene-M, hexanol-M/D, isobutanol-M, methyl butyrate-M show opposite trends. The results indicated that the volatile flavor of fermented Ciba chili could be changed by the addition of prickly ash.

**Figure 4:**
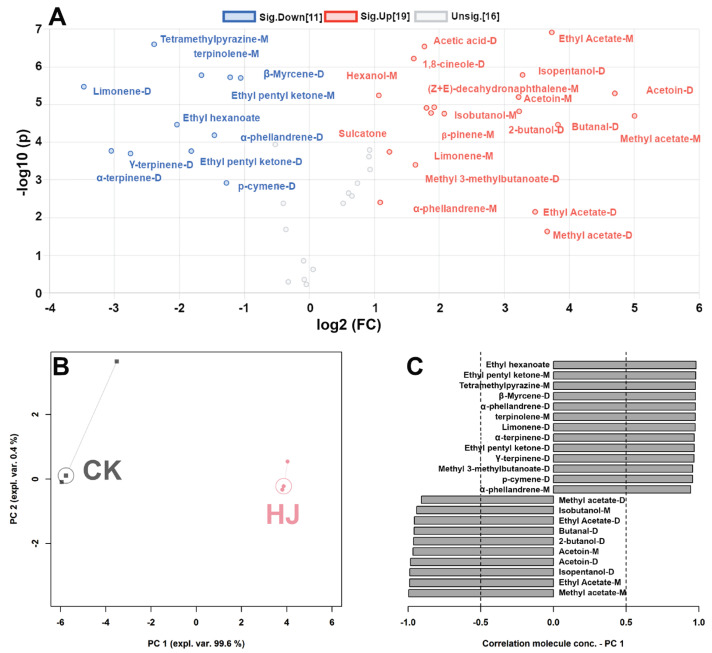
The volcano plot (**A**) illustrates the variations in the compound levels between the CK and HJ samples. Based on the molecules selected from the volcano plot (**A**), an rPCA model was established. The score plot (**B**) and Pearson correlation plot of the loadings (**C**) depict the molecules that exhibit significant correlations between concentration and importance over PC1 (*p* < 0.05).

In order to identify the molecules that show significant differences between the two types of Ciba chili, a volcano plot was constructed, combining *t*-test and fold change analyses on a per-molecule basis. The plot in Figure 4A reveals a total of 30 substances that exhibit significant differences. To gain a comprehensive understanding of the trends displayed by these molecules, their signal intensities were used to construct an rPCA model, as depicted in Figure 4B,C. Ethyl hexanoate, ethyl pentyl ketone-M, tetratmethylpyrazine-M, β-myrcene-D, α-phellandrene-M/D, terpinolene-M, limonene-D, α-terpinene-D, ethyl pentyl ketone-D, γ-terpine-D, methyl 3-methylbutanoate-D, and p-cymene-D were found to have higher concentrations in HJ. Conversely, methyl acetate-M/D, isobutanol-M, ethyl acetate-M/D, butanal-D, 2-butanol-D, acetoin-M/D, and isopentanol-D exhibited higher levels in CK.

### 2.4. Correlation between E-Nose and GC-IMS

**Figure 5:**
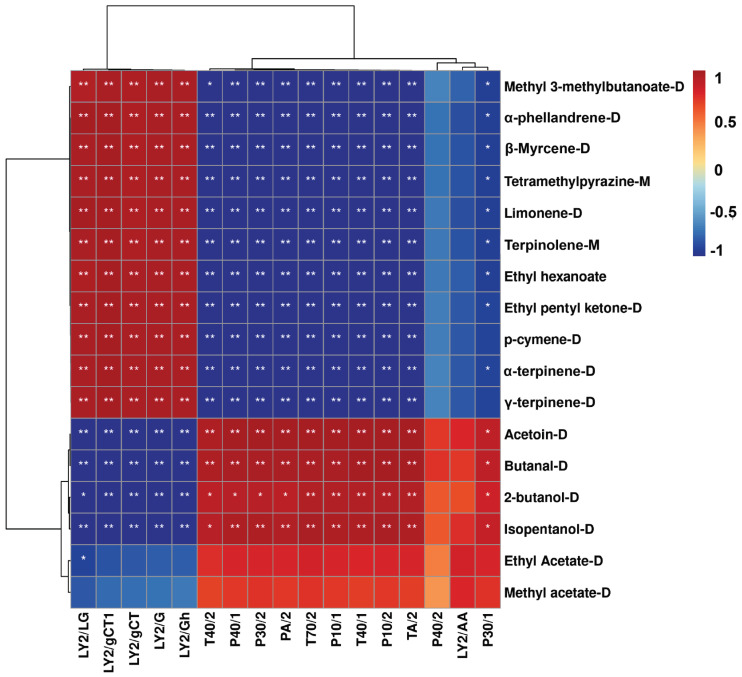
The Spearman’s correlation heatmap illustrates the correlation between the significantly altered levels of volatile compounds and the E-nose sensor responses. In the heatmap, colors represent correlation coefficients, with red indicating positive correlations and blue indicating negative correlations. ** indicates extremely significant differences (*p* < 0.01), while * denotes significant differences (*p* < 0.05).

To improve the overall effectiveness of both E-nose and GC-IMS, an investigation was conducted to explore the potential correlation between E-nose sensor responses and volatile compound levels detected by GC-IMS. As depicted in Figure 5, several sensors including LY2/LG, LY2/gCT1, LY2/gCT, LY2/G, and LY2/Gh showed a positive correlation with major compounds such as methyl 3-methylbutanoate-D, α-phellandren-D, β-myrcene-D, tetramethylpyrazine-M, limonene-D, terpinolene-M, ethyl hexanoate, ethyl pentyl ketone-D, p-cymene-D, α-terpinene-D, and γ-terpinene-D, which were identified at high levels in HJ through GC-IMS analysis. Conversely, sensors T40/2, P40/1, P30/2, PA/2, T70/2, P10/1, T40/1, P10/2, TA/2, P40/2, LY2/AA, and P30/1 exhibited a positive correlation with compounds such as acetoin-D, butanal-D, 2-butanol-D, isopentanol-D, and ethyl acetate-D, which were found to be higher in CK compared to HJ. These findings indicate that the distinct flavors of fermented Ciba chili with and without Sichuan pepper can be distinguished by analyzing the E-nose sensor response values and quantification of volatile compounds using GC-IMS.

## 3. Discussion

Flavor plays a crucial role in evaluating the quality of fermented food, and comprehending the intricate process of flavor formation necessitates a deep understanding of the diverse biochemical conversions involving food constituents. The predominant microorganisms found in fermented Ciba chili are lactic acid bacteria (LAB), encompassing both homo-fermentative and hetero-fermentative strains. These LAB possess the capability to produce lactic and acetic acids, thereby contributing to the acidity of the fermented peppers while also reducing their inherent spiciness [8]. Moreover, LAB employ key metabolic pathways such as carbohydrate metabolism, proteolysis, and amino acid metabolism, as well as lipolysis and fatty acid metabolism, to facilitate flavor development [19].

The aroma profile of fermented Ciba chili primarily consists of esters, aldehydes, alcohols, and terpenes. Among these, seventeen compounds displayed significant differences between the two sample types: α-phellandrene-D, β-myrcene-D, limonene-D, terpinolene-M, p-cymene-D, α-terpinene-D, γ-terpinene-D, tetramethylpyrazine-M, ethyl hexanoate, methyl 3-methylbutanoate-D, ethyl acetate-D, methyl acetate-D, ethyl pentyl ketone-D, acetoin-D, butanal-D, 2-butanol-D, and isopentanol-D. The inclusion of prickly ash led to a significant increase in the levels of esters and alcohols (*p* < 0.05). Esters, known for their fruity and sweet aroma characteristics, can be synthesized through various pathways, including the esterification of organic acids with alcohols, oxidation of fatty acids, or amino acid metabolism. Additionally, certain esters may be produced through alcohol-acetyl aminotransferase, which utilizes alcohol and acetyl-CoA as substrates [20]. Ethyl acetate, known for its distinct fruity flavor, was found to have higher levels in CK. Both lactic acid and acetic acid have been reported to contribute to the formation of esters, including ethyl lactate and ethyl acetate [17]. *Wickerhamomyces anomalus* has been identified as a potential enhancer of ethyl acetate production in Chinese Baijiu fermentation processes [21]. Furthermore, ethyl acetate has been frequently identified as a key flavor compound in fermented chilis [15,17,22], which is consistent with our research findings. Additionally, ethyl hexanoate is the predominant aroma compound in fermented pepper paste and gochujang products [23,24,25].

Acetoin is a pale to yellowish liquid with a pleasant yogurt aroma and a rich creamy butter flavor. It is naturally occurring and commonly used as a food additive to enhance the taste of various products [26]. Numerous microorganisms have the ability to utilize different sugars, such as glucose or sucrose, as carbon sources to produce acetoin [27]. Certain lactic acid bacteria utilize citric acid as the primary substrate for acetoin and diacetyl production [28]. Additionally, acetoin can be formed through the enzymatic condensation of two acetaldehydes [29]. The conversion of acetoin can also occur through the pyruvate derived from serine deamination [30]. Chen et al., observed that the level of acetoin increased with the progression of fermentation, and there was no statistically significant difference in its content between naturally fermented and inoculated samples [22]. Ravyts et al., reported that inoculation with *Staphylococcus sciuri α Sg2* led to acetoin production in Southern European fermented dry sausages [31]. Ethyl pentyl ketone, like many other ketones, possesses an unpleasant and pungent odor. Li et al., discovered a correlation between ethyl pentyl ketone and *Streptophyta* in bean paste [32].

During the fermentation of Ciba chili, aldehydes, which have a lower sensory threshold, contribute significantly to its flavor profile. The formation of aldehydes can be attributed to the oxidative degradation of linoleic acid and other unsaturated fatty acids, as well as the Strecker degradation of amino acids [33]. Butanal is a characteristic odor component of fermented dry chili sauce [34]. Chen et al., observed a decrease in the level of butanal during fermentation, and there was no statistically significant difference in its content between naturally fermented and inoculated samples [22].

Alcohols, characterized by botanical and fragrant aromas, are metabolic products generated by lactic acid bacteria and other microorganisms through fermentation. Their formation is primarily attributed to the thermal oxidation of lipids and the degradation of carbohydrates. Isopentanol and 2-butanol were found to be more abundant in CK, while hexanol was more prevalent in HJ.

Terpenes, which comprise a significant component of natural plants, represent the most diverse class of compounds. It is worth noting that certain fruits and vegetables may contain terpenes in a glycoconjugated form [35]. Among them, β-myrcene, with its distinct herbal note, plays a pivotal role in Chinese prickly ash [36]. Furthermore, it has been identified as a characteristic flavor compound in fermented peppers [37]. Collins et al., observed a positive correlation between *Leuconostoc* and 2,3,5,6-tetramethylpyrazine, and demonstrated that *Leuconostoc* can produce 2,3-butanediol through citric acid metabolism, which serves as an effective precursor for pyrazine synthesis [38].

During the fermentation process of Ciba chili, the addition of a specific proportion of *Zanthoxylum* has been shown to effectively increase the concentration of volatile flavor compounds, particularly terpenoids. Terpenoids are derived from isoprene and are commonly found as metabolites in plants and fungi. *Zanthoxylum* itself is rich in terpenes such as limonene, which contributes to the overall terpene content. This increase in terpene production may be attributed to the influence of *Zanthoxylum* on microorganisms, leading to enhanced terpene synthesis. Previous studies have demonstrated the inhibitory effects of *Zanthoxylum* against common pathogenic bacteria in food, including both Gram-positive and Gram-negative strains [39]. However, limited research has been conducted to analyze the impact of *Zanthoxylum* on lactic acid bacteria, especially those that dominate in Ciba chili. It is important to note that *Zanthoxylum* belongs to the spice category. Previous studies have indicated that the addition of garlic and ginger can promote the growth of lactic acid bacteria during vegetable fermentation, resulting in a reduction in microbial load [40]. Additionally, Japanese prickly ash has been found to enhance the growth of lactic acid bacteria in fermented vegetables [41]. Therefore, the influence of prickly ash on the flavor profile of Ciba chili may arise from its inherent properties or its microbial metabolites, warranting further investigation.

## 4. Materials and Methods

### 4.1. Preparation of Ciba Chili

Dried chili peppers without insect pests were carefully selected and prepared. The peppers were washed, boiled, drained, and finely minced. A mixture of 6% salt and 5% sugar was added to the minced pepper and thoroughly mixed. The samples were then divided into two groups: one group contained 5% Sichuan pepper, designated as HJ, while the other group did not contain Sichuan pepper and was referred to as CK. The prepared samples were sealed and fermented naturally in ceramic jars. After a fermentation period of 35 days, samples were collected and stored at −20 °C for further analysis.

### 4.2. E-Nose Analysis

To distinguish between different fermented Ciba chili samples, a commercial electronic nose (FOX 4000, A MOS, Toulouse, France) was employed. The E-nose consists of an injection system, 18 sensor chambers, a mass flow controller, and an acquisition board with a microcontroller. The sensors used in the E-nose and their corresponding sensitivities are as follows: PA/2, LY2/AA, P30/2, P30/1, and TA/2 are sensitive to organic compounds; LY2/LG, P40/1, P40/2, T40/2, and T40/1 are sensitive to gases with strong oxidizing capacity; LY2/G, LY2/Gh, and LY2/gCTl are sensitive to organic amines; P10/1, LY2/gCT, and P10/2 are sensitive to hydrocarbons; T30/1 is sensitive to polar compounds, while T70/2 is sensitive to aromatic compounds. For the analysis, 0.5 g of each sample was placed in a 10 mL headspace bottle and incubated at 60 °C for 5 min prior to manual injection into the E-nose. The measurement phase lasted for 120 s, followed by a rinsing phase of 240 s. Each sample was measured ten times, and five sets of stable data were recorded for further analysis.

### 4.3. E-Tongue Analysis

E-tongue analysis was conducted using the α-ASTREE instrument (A MOS, Toulouse, France), which featured a sixteen autosampler carousel for sample handling. The instrument was equipped with seven sensors for detecting sourness (AHS), saltiness (CTS), umami (NMS), sweetness (ANS), bitterness (SCS), and two reference electrodes (PKS and CPS) [11]. To extract the taste substances, 50 g of samples were mixed with 250 mL of deionized water, subjected to 30 min of ultrasonic treatment, and then filtered. An 80 mL portion of the filtrate was collected for E-tongue analysis. Each collection period lasted 120 s, with a stirring rate of 60 revolutions per minute. After each collection, a cleaning time of 30 s was implemented using deionized water as the cleaning solution. The output value was determined as the average measurement obtained between 100 and 120 s.

### 4.4. HS-GC-IMS Analysis

The headspace analysis was performed using the GC-IMS instrument (Flavourspec^®^; G.A.S. Dortmund Company, Dortmund, Germany). The instrument was equipped with a syringe and an auto-sampler unit. A capillary column, specifically an MXT-5 column (15 m × 0.53 mm ID, 1 µm), was utilized in the GC. The GC column temperature was set at 60 °C, while the IMS temperature was set at 45 °C. For sample preparation, 0.4 g of the samples were transferred into a 20 mL headspace glass sampling vial and incubated at 50 °C for 15 min. Subsequently, 200 µL of the headspace samples were automatically injected into the injector (operating at 85 °C in splitless mode) using a heated syringe. Nitrogen gas with a purity of 99.999% was employed as the carrier/drift gas, with programmed flow rates as follows: EPC1 (IMS drift gas) was maintained at 150 mL/min, while EPC2 (GC carrier gas) was initially set at 2 mL/min for 2 min and then increased to 100 mL/min within 18 min. Qualitative analysis of volatile compounds was performed based on the IMS and the NIST database integrated within the GC-IMS Library Search. As for the quantitative analysis of volatile compounds, it primarily relied on the peak intensity observed in the HS-GC-IMS, which is directly proportional to the content of the volatile compound.

### 4.5. Statistical Analysis

The samples of electronic nose and electronic tongue were analyzed in five replicates, while GC-IMS was performed in three replicates. Qualitative identification of detected compounds was performed using the GC-IMS Library Search, utilizing the built-in NIST and IMS databases. The Gallery Plot plug-in was employed to compare fingerprint patterns. Statistical analysis was conducted using the R computational language. Prior to the univariate analysis, the data distribution was normalized using the Box–Cox method. *t*-tests were utilized to determine significant differences between groups. Following the approach of previous studies, robust principal component analysis (rPCA) models were constructed using the average values of E-nose and E-tongue sensors, as well as the peak signal intensities of the molecules, in order to obtain a comprehensive overview of the data [42]. For each rPCA model, score plots and Pearson correlation plots were generated to elucidate the data structure and identify relationships between variables and model components.

## 5. Conclusions

In summary, the addition of Sichuan pepper during fermentation enhances the flavor characteristics of Ciba chili. E-tongue clearly distinguishes samples with positive responses from PKS, CTS, NMS, CPS, SCS, AHS, and ANS sensors. Consistent with the E-tongue, the E-nose findings indicate that CK exhibits a mild taste profile, while HJ displays a more intense and complex aroma. GC-IMS analysis was employed to characterize and identify flavor compounds, resulting in the selection of 17 distinct compounds through rPCA. The correlation analysis between E-nose sensors and flavor compounds reveals that most terpenes show positive correlations with LY2/LG, LY2/gCT1, LY2/gCT, LY2/G, and LY2/Gh. These terpenes are present in higher quantities in HJ. Furthermore, the integration of electronic tongue, electronic nose, and headspace gas chromatography–ion mobility spectrometry effectively distinguishes the presence of Sichuan pepper flavor attributes in Ciba chili. In future studies, it would be worthwhile to investigate the effects of different quantities and types of Sichuan pepper, as well as combinations with other spices, on the flavor profile of Ciba chili. This exploration aims to optimize the sensory attributes of Ciba chili for industrial production.

## Figures and Tables

**Table 1:** The peak areas (mean ± sd) of molecules characterized by GC–IMS in fermented Ciba chili samples from CK and HJ are presented.

Count	Compound	CAS	Molecule Formula	MW	RI	Rt [sec]	Dt [a.u.]	CK	HJ	*p*-Value
Alcohols	1,8-cineole-D	470-82-6	C_10_H_18_O	154	1223	776.99	1.7295	610.93 ± 38.94	1031.96 ± 341.23	0.036
	1,8-cineole-M	470-82-6	C_10_H_18_O	154	1226	787.731	1.29699	2661.02 ± 46.46	9163.40 ± 1265.95	0.030
	Hexanol-D	111-27-3	C_6_H_14_O	102	1331	1126.15	1.62945	490.40 ± 24.12	1340.22 ± 45.36	0.180
	Hexanol-M	111-27-3	C_6_H_14_O	102	1330	1123.04	1.32811	1919.54 ± 28.09	3286.81 ± 25.02	<0.000
	pentan-1-ol-D	71-41-0	C_5_H_12_O	88.1	1268	928.815	1.51635	63.52 ± 6.57	210.17 ± 19.93	0.031
	pentan-1-ol-M	71-41-0	C_5_H_12_O	88.1	1266	921.21	1.25183	806.66 ± 27.75	4480.32 ± 65.23	<0.000
	2-butanol-D	78-92-2	C_4_H_10_O	74.1	1027	379.352	1.3485	1038.69 ± 16.06	580.54 ± 102.37	<0.000
	2-butanol-M	78-92-2	C_4_H_10_O	74.1	1026	377.748	1.13051	638.03 ± 33.08	354.58 ± 122.76	<0.000
	Isobutanol-M	78-83-1	C_4_H_10_O	74.1	1106	479.891	1.16782	1226.29 ± 31.54	1520.15 ± 131.51	<0.000
	Isobutanol-D	78-83-1	C_4_H_10_O	74.1	1106	479.499	1.36851	16,440.99 ± 268.83	14,455.76 ± 680.24	<0.000
	Isopentanol-D	123-51-3	C_5_H_12_O	88.1	1221	770.767	1.52072	4364.86 ± 236.67	2339.17 ± 200.46	<0.000
	Isopentanol-M	123-51-3	C_5_H_12_O	88.1	1226	786.33	1.2482	756.99 ± 57.58	737.57 ± 92.66	<0.000
	1-Butanol-D	71-36-3	C_4_H_10_O	74.1	1152	584.648	1.38006	175.17 ± 13.46	1173.69 ± 93.19	<0.000
	1-Butanol-M	71-36-3	C_4_H_10_O	74.1	1152	584.862	1.18174	488.67 ± 16.30	1352.72 ± 117.09	0.094
Aldehydes	Butanal-D	123-72-8	C_4_H_8_O	72.1	849	259.374	1.27451	602.52 ± 41.57	221.66 ± 39.03	<0.000
	Butanal-M	123-72-8	C_4_H_8_O	72.1	853	261.669	1.11185	2834.40 ± 104.63	3084.93 ± 101.04	<0.000
Acids	Acetic acid-D	64-19-7	C_2_H_4_O_2_	60.1	1521	1695.04	1.15617	2178.07 ± 96.07	3329.42 ± 118.37	<0.000
	Acetic acid-M	64-19-7	C_2_H_4_O_2_	60.1	1522	1697.23	1.06686	9937.87 ± 320.84	10,760.98 ± 133.24	<0.000
Terpenes	α-phellandrene-D	99-83-2	C_10_H_16_	136	1126	526.249	1.61985	296.67 ± 33.90	4271.71 ± 140.15	<0.000
	α-phellandrene-M	99-83-2	C_10_H_16_	136	1127	527.454	1.22194	2697.31 ± 104.86	6705.72 ± 269.55	0.134
	α-pinene-D	80-56-8	C_10_H_16_	136	1027	379.543	1.29874	39.84 ± 1.58	145.34 ± 15.36	0.009
	α-pinene-M	80-56-8	C_10_H_16_	136	1027	379.779	1.21667	454.99 ± 9.25	1250.16 ± 87.74	0.108
	α-terpinene-D	99-86-5	C_10_H_16_	136	1185	657.491	1.21571	126.54 ± 14.23	5471.64 ± 626.21	<0.000
	α-terpinene-M	99-86-5	C_10_H_16_	136	1186	659.873	1.72906	2054.02 ± 98.48	10,301.88 ± 56.55	<0.000
	β-pinene-D	127-91-3	C_10_H_16_	136	1115	499.51	1.29743	105.52 ± 3.11	582.62 ± 69.11	0.001
	β-pinene-M	127-91-3	C_10_H_16_	136	1115	499.8	1.21209	1262.44 ± 39.81	1800.12 ± 140.29	<0.000
	γ-terpinene-D	99-85-4	C_10_H_16_	136	1253	876.66	1.7029	151.56 ± 21.35	5327.60 ± 612.07	<0.000
	γ-terpinene-M	99-85-4	C_10_H_16_	136	1253	878.248	1.21571	1899.98 ± 124.29	14248.92 ± 133.09	<0.000
	Limonene-D	138-86-3	C_10_H_16_	136	1203	709.944	1.65473	58.24 ± 3.51	3380.48 ± 102.67	<0.000
	Limonene-M	138-86-3	C_10_H_16_	136	1200	700.748	1.22221	882.37 ± 47.61	1319.73 ± 52.80	<0.000
	β-Myrcene-D	123-35-3	C_10_H_16_	136	1172	628.655	1.2899	420.17 ± 22.04	4562.3 ± 109.59	<0.000
	β-Myrcene-M	123-35-3	C_10_H_16_	136	1170	625.242	1.21212	3370.93 ± 159.65	3913.98 ± 124.57	<0.000
	terpinolene-D	586-62-9	C_10_H_16_	136	1290	1002.66	1.30375	173.27 ± 15.61	1192.91 ± 89.14	<0.000
	terpinolene-M	586-62-9	C_10_H_16_	136	1290	1001.71	1.22495	293.61 ± 24.22	4844.98 ± 108.2	<0.000
Ketones	2,3-Butanedione	431-03-8	C_4_H_6_O_2_	86.1	939	311.167	1.16367	5013.84 ± 39.84	5001.38 ± 99.22	<0.000
	2,5-Dimethylfuran	625-86-5	C_6_H_8_O	96.1	911	295.279	1.34045	27,492.09 ± 198.29	24,734.86 ± 1697.27	<0.000
	Butanone-D	78-93-3	C_4_H_8_O	72.1	839	253.555	1.22842	140.76 ± 50.27	172.99 ± 9.04	0.033
	Butanone-M	78-93-3	C_4_H_8_O	72.1	839	253.831	1.07074	726.38 ± 148.78	642.16 ± 30.99	0.006
	Acetoin-D	513-86-0	C_4_H_8_O_2_	88.1	1299	1031.38	1.33746	3672.04 ± 264.32	736.92 ± 119.94	<0.000
	Acetoin-M	513-86-0	C_4_H_8_O_2_	88.1	1298	1027.43	1.06717	1456.46 ± 37.25	814.16 ± 207.12	<0.000
	Ethyl pentyl ketone-D	106-68-3	C_8_H_16_O	128	1245	850.262	1.71143	231.05 ± 15.04	4251.33 ± 392.19	<0.000
	Ethyl pentyl ketone-M	106-68-3	C_8_H_16_O	128	1245	851.414	1.27983	556.61 ± 14.49	6765.04 ± 260.43	<0.000
	Sulcatone	110-93-0	C_8_H_14_O	126	1348	1177.16	1.17719	215.75 ± 3.62	531.65 ± 176.28	0.025
Esters	n-Butyl acetate-D	123-86-4	C_6_H_12_O_2_	116	1087	448.621	1.61924	29.36 ± 1.13	191.52 ± 90.97	0.018
	n-Butyl acetate-M	123-86-4	C_6_H_12_O_2_	116	1088	449.398	1.23959	58.49 ± 1.94	316.07 ± 32.48	0.001
	Ethyl Acetate-D	141-78-6	C_4_H_8_O_2_	88.1	878	276.381	1.33139	259.48 ± 85.65	121.17 ± 7.7	0.012
	Ethyl Acetate-M	141-78-6	C_4_H_8_O_2_	88.1	883	279.189	1.08464	1796.44 ± 77.15	705.51 ± 40.1	<0.000
	Ethyl hexanoate	123-66-0	C_8_H_16_O_2_	144	1211	736.541	1.8022	144.80 ± 12.81	3103.68 ± 246.22	<0.000
	Methyl 3-methylbutanoate-D	556-24-1	C_6_H_12_O_2_	116	1020	371.709	1.54279	142.58 ± 8.56	317.14 ± 22.6	0.048
	Methyl 3-methylbutanoate-M	556-24-1	C_6_H_12_O_2_	116	1015	365.186	1.20431	2047.52 ± 33.27	1528.09 ± 276.3	<0.000
	Methyl acetate-D	79-20-9	C_3_H_6_O_2_	74.1	789	225.283	1.20525	548.69 ± 254.41	224.77 ± 3.77	0.034
	Methyl acetate-M	79-20-9	C_3_H_6_O_2_	74.1	790	225.645	1.04573	2018.83 ± 181.67	327.69 ± 26.57	<0.000
	Methyl butyrate-D	623-42-7	C_5_H_10_O_2_	102	969	328.537	1.45813	3620.72 ± 183.59	5145.61 ± 1010.13	0.003
	Methyl butyrate-M	623-42-7	C_5_H_10_O_2_	102	968	327.908	1.13301	2987.22 ± 130.77	4106.43 ± 675.94	0.001
Others	(Z + E)-decahydronaphthalene-D	91-17-8	C_10_H_18_	138	1153	586.251	1.34113	1224.9 ± 98.15	981.59 ± 301.69	0.001
	(Z + E)-decahydronaphthalene-M	91-17-8	C_10_H_18_	138	1154	587.484	1.26016	736.92 ± 16.3	1013.44 ± 117.03	<0.000
	p-cymene-D	99-87-6	C_10_H_14_	134	1264	916.29	1.70737	740.06 ± 92.73	9355.16 ± 1067.88	<0.000
	p-cymene-M	99-87-6	C_10_H_14_	134	1257	892.069	1.30337	335.84 ± 26.64	1050.78 ± 95.45	0.039
	Tetramethylpyrazine-D	1124-11-4	C_8_H_12_N_2_	136	1458	1506.71	1.67224	1713.86 ± 79.93	12,582.39 ± 712.91	<0.000
	Tetramethylpyrazine-M	1124-11-4	C_8_H_12_N_2_	136	1448	1478.24	1.22068	3724.63 ± 174.51	101,844.89 ± 1652.83	<0.000

Notes: MW—molecular mass; RI—retention index; Rt—retention time; Dt—drift time. *p*-value was calculated by *t*-test, and the cutoff value was set as below 0.05.

## Data Availability

Not applicable.
